# Importance of the Long-Chain Fatty Acid Beta-Hydroxylating Cytochrome P450 Enzyme YbdT for Lipopeptide Biosynthesis in *Bacillus subtilis* Strain OKB105

**DOI:** 10.3390/ijms12031767

**Published:** 2011-03-08

**Authors:** Noha H. Youssef, Neil Wofford, Michael J. McInerney

**Affiliations:** 1 Department of Microbiology and Molecular Genetics, Oklahoma State University, Stillwater, OK 74078, USA; 2 Department of Botany and Microbiology, University of Oklahoma, Norman, OK 74078, USA; E-Mails: nqw@ou.edu (N.W.); mcinerney@ou.edu (M.J.M.)

**Keywords:** cytochrome P450, YbdT, long chain fatty acids, beta hydroxylation, *Bacillus subtilis*, surfactin

## Abstract

*Bacillus* species produce extracellular, surface-active lipopeptides such as surfactin that have wide applications in industry and medicine. The steps involved in the synthesis of 3-hydroxyacyl-coenzyme A (CoA) substrates needed for surfactin biosynthesis are not understood. Cell-free extracts of *Bacillus subtilis* strain OKB105 synthesized lipopeptide biosurfactants in presence of l-amino acids, myristic acid, coenzyme A, ATP, and H_2_O_2_, which suggested that 3-hydroxylation occurs prior to CoA ligation of the long chain fatty acids (LCFAs). We hypothesized that YbdT, a cytochrome P450 enzyme known to beta-hydroxylate LCFAs, functions to form 3-hydroxy fatty acids for lipopeptide biosynthesis. An in-frame mutation of *ybdT* was constructed and the resulting mutant strain (NHY1) produced predominantly non-hydroxylated lipopeptide with diminished biosurfactant and beta-hemolytic activities. Mass spectrometry showed that 95.6% of the fatty acids in the NHY1 biosurfactant were non-hydroxylated compared to only ∼61% in the OKB105 biosurfactant. Cell-free extracts of the NHY1 synthesized surfactin containing 3-hydroxymyristic acid from 3-hydroxymyristoyl-CoA at a specific activity similar to that of the wild type (17 ± 2 *versus* 17.4 ± 6 ng biosurfactant min^−1^·ng·protein^−1^, respectively). These results showed that the mutation did not affect any function needed to synthesize surfactin once the 3-hydroxyacyl-CoA substrate was formed and that YbdT functions to supply 3-hydroxy fatty acid for surfactin biosynthesis. The fact that YbdT is a peroxidase could explain why biosurfactant production is rarely observed in anaerobically grown *Bacillus* species. Manipulation of LCFA specificity of YbdT could provide a new route to produce biosurfactants with activities tailored to specific functions.

## 1. Introduction

Biosurfactants are surface-active agents produced by a wide variety of microorganisms that partition at the water/air and water/oil interfaces [1]. By partitioning at these interfaces, biosurfactants can lower the surface and interfacial tensions between the two phases [1–4] and hence have a wide variety of applications in industry [1–4] and medicine [5].

Biosurfactants have diverse chemical structures including glycolipids, phospholipids, and lipopeptides [3]. One of the most studied types of biosurfactants is lipopeptides produced by *Bacillus* spp. Most lipopeptide biosurfactants have been shown to have a structure similar to that of surfactin, the biosurfactant produced by *Bacillus subtilis* [6–9]. Surfactin as well as other lipopeptides produced by *Bacillus* spp. are synthesized non-ribosomally through the action of non-ribosomal peptide synthetases (NRPS), which are large multifunctional enzymes with a modular organization of condensation, adenylation, and thiolation domains [10–14]. The activity of surfactinsynthetase (SrfABC) has been studied in detail over the past two decades [15–21]. Recently, it was shown that in the initiation reaction, the 3-hydroxy fatty acid substrate is transferred from coenzyme A (CoA) to SrfA, where 3-hydroxyacyl-glutamate is formed [22]. SrfD, the external thioesterase enzyme in surfactin biosynthesis, was shown to stimulate the formation of the initiation product [22]. Kraas *et al.* [23] showed that the condensation domain of the initiation module catalyzed the transfer of the CoA-activated 3-hydroxy long chain fatty acid (LCFA) to the peptidyl carrier protein-bound glutamate. They also showed that the activation of 3-hydroxy LCFA occurs via the activity of two acyl CoA ligases in *Bacillus subtilis* [23]. However, the reaction involved in the formation of the 3-hydroxy-fatty acids needed for initiation of biosurfactant synthesis is not known.

Previous work showed that changes in the amino acid and fatty acid composition of lipopeptides had a pronounced effect on lipopeptide activity [24–29]. Surfactin with a hydrophobic pentadecanoic fatty acid side chain was found to be the most active with regards to hemolytic activity [28]. Also, increasing the percentage of *iso*-even-numbered fatty acids compared to *n*-even-numbered fatty acids in the lipopeptide biosurfactant increased the activity of the lipopeptide as determined by the oil spreading assay [29]. The identification of the enzyme responsible for 3-hydroxylation of LCFAs involved in biosurfactant synthesis could potentially provide a new route for manipulation of biosurfactant activity. By increasing the specificity of this enzyme towards branched *versus* straight chain and even- *versus* odd-numbered fatty acids, biosurfactants with a tailored mixture of 3-hydroxy fatty acyl moieties could be produced to suit the required task.

Cytochrome P450 enzymes are known to hydroxylate LCFAs in *Bacillus* sp. [30,31]. *B. subtilis* genome has genes for eight cytochrome P450 enzymes [30]. BioI (CYP107H1) catalyzes in-chain cleavage of fatty acids bound to ACP as part of pimelic acid formation during biotin biosynthesis, but can catalyze sub-terminal hydroxylation of LCFAs *in vitro* [32,33]. CYP102A2 and CYP102A3, the *B. subtilis* homologues of the flavocytochrome CYP102A1 from *Bacillus megaterium*, catalyze the sub-terminal hydroxylation of long chain unsaturated and branched chain fatty acids in the presence of heme, FAD, and FMN as cofactors [34]. PksS is involved in hydroxylation of polyketides [35], YjiB is proposed to be involved in the catabolism of hexuronate [36], CypA is proposed to be involved in the transport of branched-chain amino acids [37], and CypX (CYP134A1) is involved in the three-step, oxidative transformation of the diketopiperazinecyclo-l-leucyl-l-leucyl to pulcherriminic acid and has recently been recognized as a cyclo-l-leucyl-l-leucyldipeptideoxidase [38]. Only one cytochrome P450 enzyme, YbdT (CYP152A1), is known to hydroxylate LCFAs in the α/β position [39,40].

We mutated *ybdT* (CYP152A1) to test the role of YbdT in the formation of 3-hydroxy-LCFAs needed for lipopeptide biosurfactant synthesis. Manipulation of the YbdT active site to accommodate different fatty acids is discussed with reference to its potential for enhancing biosurfactant surface activity.

## 2. Results

### 2.1. In-Frame Mutation of *ybdT* in *B. subtilis* Strain OKB105

**Figure 1.**
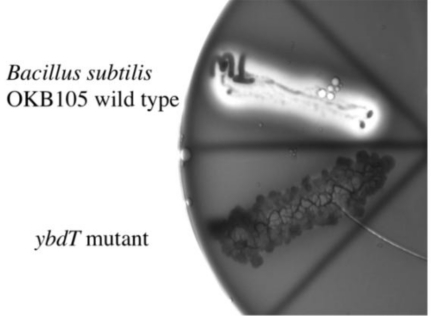
β-hemolytic activity of the OKB105 cells compared to NHY1, the *ybdT* mutant. Blood agar plate was streaked with a single colony of OKB105 (wild-type) or NHY1 (*ybdT* mutant) cells. β-Hemolysis occurred within 24 h in OKB105 cells with clearing of blood agar and the appearance of a green sheen. NHY1 cells showed delayed hemolysis, which occurred after 48 h of incubation, and was not as extensive as that observed with OKB105 cells. The picture was taken 24 h after streaking.

The initiation of surfactin synthesis involves the transfer of the 3-hydroxyacyl moiety from 3-hydroxyacyl-CoA to SrfA [22]. Whether the 3-hydroxyacyl moiety of surfactin is derived from 3-hydroxyacyl-CoA generated in the beta-oxidation pathway or from the 3-hydroxylation of LCFAs is not known. We hypothesized that the LCFA 3-hydroxylating enzyme, YbdT, is responsible for the 3-hydroxylation of the fatty acids used for surfactin synthesis. To test this hypothesis, we mutated the *ybdT* via transformation of competent *B. subtilis* OKB105 cells with a PCR construct carrying a chloramphenicol-resistance cassette in-frame with the *ybdT* gene sequence. The competence of OKB105 was checked with a PCR product of *rpoB* gene that confers resistance to rifampicin. The percent transformation of OKB105 cells with *rpoB* gene was 2.05 × 10^−4^, comparable to a percent transformation of 7.7 × 10^−4^ reported previously [41]. A chloramphenicol-resistant mutant with delayed hemolysis of blood agar (NHY1) was obtained as a potential *ybdT*^−^ mutant (Figure 1). Amplification and sequencing of the *ybdT* gene from NHY1 showed that the chloramphenicol-resistance cassette was inserted into the *ybdT* gene, confirming that NHY1 was a *ybdT* mutant.

### 2.2. YbdT Activity of OKB105 and NHY1 Cells

**Figure 2.**
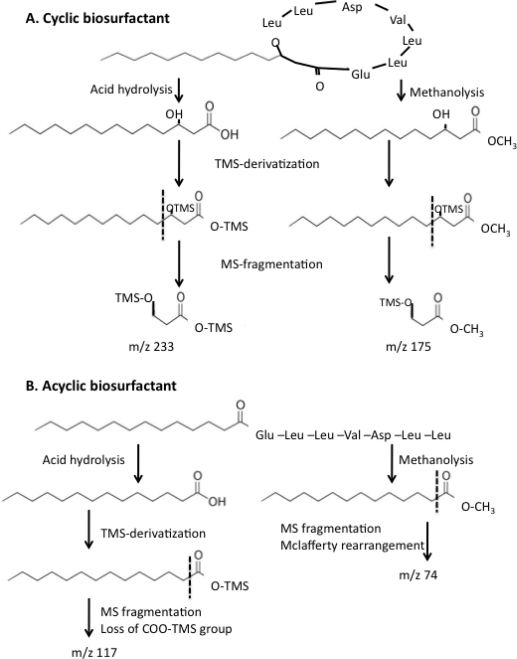
Summary of the outcome of acid hydrolysis and methanolysis of (**A**) a cyclic lipopeptide biosurfactant, and (**B**) an acyclic (linear) lipopeptide biosurfactant. Each type of biosurfactant was either acid hydrolyzed or methanolyzed (as described in text) with the release of a free fatty acid, or a fatty acid methyl ester (FAME), respectively. The fatty acid and FAME were then derivatized, separated by gas chromatography, and identified by mass spectrometry. Dotted line refers to the fragmentation pattern of the mass ion used to extract the MS chromatogram as explained in text.

YbdT is a peroxygenase that requires hydrogen peroxide for catalysis and its activity can be followed by the formation of 3-hydroxyl fatty acids from LCFA in the presence of H_2_O_2_ [39]. No myristic acid was detected by gas chromatography-mass spectroscopy (GC/MS) after OKB105 cell-free extracts incubated in the presence of myristic acid were acid precipitated and derivatized with N,O-bis(trimethylsilyl)trifluoroacetamide (BSTFA). The GC chromatogram showed a peak that had the same retention time and fragmentation pattern as bis-trimethylsilyl (TMS)-derivatized 3-hydroxymyristic acid standard. The reaction mixture with the NHY1 cell-free extract contained only a peak for the TMS-derivative of myristic acid, and no peaks were detected upon extraction of the MS chromatogram at *m/z* of 233 (see Figure 2a for description of the mass spectrometry fragmentation of bis-TMS-derivatized 3-hydroxymyristic acid), indicating the absence of 3-hydroxy fatty acids. The absence of a hydroxylated fatty acid in NHY1 extracts confirmed that YbdT is a 3-hydroxylating LCFA peroxidase.

Cell free extracts of OKB105 synthesized surfactin in the presence of the free fatty acid, CoA, and H_2_O_2_ with a specific activity of 31 ng biosurfactant·min^−1^·ng·protein^−1^, which is comparable to the rate when 3-hydroxymyristoyl-CoA was used (30 ng biosurfactant·min^−1^·ng·protein^−1^).

### 2.3. Effect of the *ybdT* Gene Mutation on Surfactin Synthesis

NHY1 cultures had reduced oil-displacement activity (0.5 cm) compared to OKB105 cultures (2 cm). Upon purification, the NHY1-biosurfactant had a specific activity of 0.5 mm of oil displacement per μg compared to 2.8 mm per μg of OKB105 biosurfactant [29]. To rule out the possibility that the low biosurfactant activity of NHY1 was due to lack of expression of the *srfABC* operon, *srf* gene transcription in NHY1 was investigated with reverse transcriptase PCR. A band of the expected size (250 bp) was obtained with RNA extracted from NHY1 and OKB105 strains indicating that the surfactin synthesis gene was expressed in presence of the *ybdT* gene mutation.

As shown above, NHY1 did not hydroxylate LCFA. However, in the presence of 3-hydroxyacyl-CoA, NHY1 should be able to synthesize surfactin at a rate comparable to that of OKB105. To test this hypothesis, biosurfactant synthesis activity was determined in three ways (Table 1). There was no significant difference between cell-free extracts of NHY1 and OKB105 in the rate of biosurfactant formation as determined by the disappearance of 3-hydroxymyristoyl-CoA, the release of CoA, and the formation of surfactin. These data show that the *ybdT* mutation had no effect on surfactin synthase activity when 3-hydroxymyristoyl-CoA was supplied.

### 2.4. Effect of *ybdT* Gene Mutation on Biosurfactant Structure

Since the *ybdT* gene mutation did not affect the enzyme activity of surfactin synthase, we hypothesized that the *ybdT* mutation resulted in a biosurfactant with an altered structure, one with little to no 3-hydroxyfatty acids and thus less activity. Biosurfactants were purified from NHY1 and OKB105 cultures. Amino acid analysis showed that the purified biosurfactants produced by NHY1 and OKB105 had the same amino acid composition: 1 Glu(n): 1 Asp(n): 1 Val: 4 Leu.

The non-polar fatty acid tail was analyzed by GC/MS after methanolysis and acid hydrolysis followed by derivatization with BSTFA (Figure 2). Acid hydrolysis and methanolysis, cyclic biosurfactants yield 3-hydroxy fatty acids and 3-hydroxy fatty acid methyl esters, respectively, which, after derivatization with BSTFA and MS fragmentation, will yield characteristic *m/z* ions of 233 and 175, respectively (Figure 2A). In contrast, acid hydrolysis and methanolysis of acyclic biosurfactants generate fatty acids or fatty acid methyl esters, respectively (Figure 2B). Only the former is derivatized with BSTFA and MS fragmentation yields characteristic *m/z* ions of 117 and 74, respectively (Figure 2B) (see the American Oil Chemist’s Society’s lipid library archive [43–45], and Reference [46] for published procedures and spectra).

Following methanolysis, 3-hydroxylated fatty acids methyl esters (FAME) of chain lengths of C13, C14, and C15 comprised 33.4% of total FAME detected in the OKB105 biosurfactant. Non-hydroxylated FAME with a chain length of C13–C18 accounted for 66.6% of the total FAME in the OBK105 biosurfactant. The NHY1 biosurfactant, on the other hand, had only 6.7% of the total FAME as 3-hydroxylated FAME, which had chain lengths of C13–C15, while the overwhelming majority of FAME (93.3%) were non-hydroxylated and had chain lengths of C12, C13, C14, C16, and C18 (Table 2).

Analysis of fatty acid composition of the OKB105 and NHY1 biosurfactants after acid hydrolysis and BSTFA derivatization confirmed that the NHY1 biosurfactant had a very low percentage of 3-hydroxylated fatty acids (Table 2). 3-Hydroxylated fatty acids with chain lengths of C13–C15 comprised 44.6% of total fatty acids of the OKB105 biosurfactant. Non-hydroxylated fatty acids with chain lengths of C14–C18 accounted for 55.4% of the total fatty acids in the OKB105 biosurfactant. NHY1 biosurfactant had 3-hydroxylated fatty acids with chain lengths of C14 that comprised only 2.2% of the total fatty acids, while the majority of fatty acids (97.8%) were non-hydroxylated and had chain lengths of C12, and C14–C18 (Table 2).

The molecular weights of OKB105 and NHY1 biosurfactants were determined by liquid chromatography-mass spectrometry (LC/MS) [7,47–49] (Table 3). The biosurfactants were dissolved under conditions in which the lactone ring should remain intact (room temperature in 10 mM NH_4_OH). Under these conditions, a difference in molecular weight of +2 units corresponds to the absence of the 3-hydroxyl group. OKB105 biosurfactant had peaks corresponding to 3-hydroxy fatty acids ranging from 11 to 16 carbons (Table 3). Three peaks of lower intensities were detected that corresponded to non-hydroxylated fatty acids with 12 and 13 carbons. The presence of non-hydroxylated acyclic structures in the biosurfactant produced by *Bacillus* sp. has been reported before [50]. The NHY1 biosurfactant had peaks corresponding to molecules with non-hydroxylated fatty acids with 11, 12, 13, 14, and 16 carbons (Table 3). Three peaks with lower intensities corresponding to 3-hydroxy fatty acids with 12, 13, and 14 carbons were detected.

## 3. Discussion

Steller *et al.* [22] showed that 3-hydroxymyristoyl-CoA is transferred from CoA to SrfA to initiate lipopeptide biosynthesis. The origin of 3-hydroxyacyl-CoA needed for lipopeptide biosurfactant synthesis was not known. OKB105 cell-free extracts synthesized lipopeptide biosurfactants in the presence of myristic acid, CoA, and H_2_O_2_, which suggested that 3-hydroxylation preceded CoA ligation of the LCFA used for biosurfactant synthesis. Our data provide convincing evidence that YbdT functions to 3-hydroxylate LCFA for lipopeptide biosurfactant synthesis. NHY1 had diminished hemolytic and biosurfactant activity. Over 90% of the fatty acids of the NHY1 biosurfactant were non-hydroxylated compared to 60% non-hydroxylated fatty acids in the OKB105 biosurfactant (Table 3). NHY1 made the wild-type biosurfactant only in the presence of 3-hydroxymyristoyl-CoA, while OKB105 made biosurfactant from both 3-hydroxymyristoyl-CoA and myristic acid, H_2_O_2_, and CoA.

Our PCR analyses indicated that the only difference between the OKB105 and NHY1 is the *ybdT* gene interruption. However, we were unable to introduce the wild-type gene into NHY1 to prove that disruption of this gene alone caused the NHY1 phenotype. Despite this, our studies show that NHY1 can synthesize active biosurfactant if 3-hydroxylacyl-CoA substrates are added to cell-free extract (Table 1). Thus, it seems reasonable to conclude that YbdT is required to hydroxylate LCFA for use in biosurfactant biosynthesis.

The greatly diminished amount of 3-hydroxyl fatty acids in NHY1 biosurfactant would explain its lower surface activity compared to the OKB105 biosurfactant. Without a hydroxyl group in the 3-position, the lactonization or cyclization of the biosurfactant would not be possible. Saponification of surfactin to form a linear structure changed its tertiary structure due to the partial topological disorder of the hydrophilic carboxyl group and the hydrophobic fatty acyl group [51]. The oil displacement activity of linear surfactin was only one-third of the cyclic form. We found that the NHY1 biosurfactant activity was about one-fourth of the OKB105 biosurfactant.

Previously, we found that many *B. subtilis* strains that produced biosurfactants aerobically, did not show any surface activity when grown under strict anaerobic conditions [29]. Possible explanations for this observation could be that the *ybdT* gene is not expressed or YbdT is not functional under anaerobic conditions. YbdT is a cytochrome P450 enzyme that requires H_2_O_2_ for its function. H_2_O_2_ would be made only when oxygen contamination occurred. Thus, YbdT, if present, would not be active under anaerobic growth conditions. If surfactin synthase was made anaerobically, it would make lipopeptides with non-hydroxylated fatty acids, which would form a linear lipopeptide with much lower surface activity than the cyclic counterpart [51]. The activity of any linear biosurfactants may have been too low to be detected when biosurfactant activity of culture fluids are screened by drop collapse or oil-spreading assays.

Previous work showed that surface activity of lipoheptapeptides with structures analogous to surfactin increased when the ratio of *iso-* to *normal*-even-numbered long chain fatty acids in the non-polar tail increased [29]. The identification of the enzyme responsible for the 3-hydroxylation of LCFA may provide a new route to optimization of biosurfactant surface activity by manipulation of the substrate specificity of the 3-hydroxylating enzyme. The substrate-binding pocket of YbdT has been identified [40]. Two types of interactions are involved: hydrophobic interactions with the fatty acid side chain and ten residues of YbdT and electrostatic interactions with the carboxyl group of the fatty acid with Arg242 [40]. Site-directed mutagenesis of the ten amino acids involved in the hydrophobic interactions with the alkyl side chain could yield enzymes with altered specificity towards branched-chain and longer chain fatty acid tails. It may be possible then to tailor the activity of the biosurfactant to different kinds of oils or for other uses by making biosurfactants with different fatty acid compositions.

## 4. Experimental Section

### 4.1. Strains, Plasmids and Growth Conditions

*B. subtilis* strain OKB105 was obtained from the *Bacillus* Genetic Stock Center (BGSCID:1A698). Rifampicin-resistant *Bacillus mojavenesis* strain JF-2 was obtained by heavily streaking the rifampicin-sensitive strain on LB plates with 10 μg/mL rifampicin and incubation at 37 °C for several days until growth occurred. Rifampicin resistance is conferred by a spontaneous mutation in cluster I of *rpo*B gene resulting in amino acid replacements: Q469R, H482R, H482Y, or S487L [52,53]. *Escherichia coli* harboring the chloramphenicol acetyl transferase (*cat*)-encoding plasmid PDG1662 was obtained from the *Bacillus* Genetic Stock Center (BGSCID:ECE113).

*B. subtilis* strain OKB105 was grown in minimal medium E [29] at 37 °C for 40–44 h without shaking. Cells were then harvested and used for RNA extraction or cell-free extract preparation as described previously [54]. The cell-free supernatant was used for biosurfactant purification [29]. Blood agar plates [55] were used for secondary screening of mutants for biosurfactant production because surfactin-producing strains are known to be β-hemolytic [6].

### 4.2. PCR Construct Preparation

**Figure 3.**
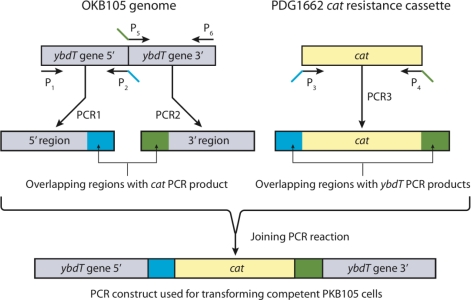
The overlap PCR protocol used to generate a *ybdT* mutation in OKB105. Primers P1 and P2 amplified the 5′ region of *ybdT* gene in OKB105. Primers P5 and P6 amplified the 3′ region of *ybdT* gene. Primers P3 and P4 amplified the chloramphenicol resistance cassette from PDG1662. Primers P2 P3, P4 and P5 were designed with 18 bp overlap as shown in Table 4. PCR1, PCR2, and PCR3 are the primary PCR products with overlapping region as follows: the 3′end of PCR1 contains sequence for the upstream portion of the *cat* and the 5′ end of PCR3 has sequence for amino acids 149–151 encoded by *ybdT* (blue). The 5′end of PCR2 contains sequence for amino acids 214–226 encoded by *cat* and 3′end of PCR3 has sequence for amino acids 365–367 encoded by *ybdT* (red). The primary PCR products were then joined in a long PCR reaction to synthesize the PCR construct used for transforming competent OKB105 cells.

An overlap PCR approach [56–58] was used to create a PCR construct of *ybdT* gene with a *cat* gene insertion (Figure 3).

DNA was extracted from *B. subtilis* OKB105 cells grown in medium E by using QIAamp DNA Mini Kit (Qiagen^®^, Valencia, CA, USA). *E. coli* harboring PDG1662 plasmid was grown according to manufacturer’s instructions and plasmid DNA was extracted using Plasmid Mini Kit (Qiagen^®^, Valencia, CA, USA). Six primers were utilized to create three PCR products (Table 4), which were then joined in the overlap PCR reaction (Figure 2).

All PCR reactions were conducted in 50 μL volume. The reaction contained 2 μL of OKB105 or PDG1662 DNA, 1 × PCR buffer with magnesium sulfate (Roche^®^, Boulder, CO, USA), 0.2 mM dNTPs mixture, 0.5 U of the Expand Long Template PCR System Taq polymerase (Roche^®^, Boulder, CO, USA), and 10 μM each of the forward and the reverse primers. The PCR reaction was carried out according to the following protocol: initial denaturation at 95 °C for 5 min, followed by 30 cycles of denaturation at 95 °C for 1 min, annealing at 65 °C for 1 min, and elongation at 72 °C for 1 min. A final elongation step at 72 °C for 5 min was included. For *cat* PCR, the number of cycles was increased to 35 and the annealing temperature was 50 °C. PCR products of the 5′ *yb*dT, 3′ *ybdT*, and *cat* were purified using MinElute PCR purification kit (Qiagen^®^, Valencia, CA, USA). The purified products were quantified spectrophotometrically. To create the PCR construct by the overlap PCR protocol, each reaction contained 0.3 μg of each purified PCR product, 1 × PCR buffer with magnesium sulfate (Roche^®^, Boulder, CO, USA), 0.2 mM dNTPs mixture, and 0.5 U of the Expand Long Template PCR System Taq polymerase (Roche^®^, Boulder, CO, USA). The PCR protocol used was an initial denaturation at 95 °C for 5 min followed by 15 cycles of denaturation at 95 °C for 30 s, annealing at 55 °C for 30 s, and elongation at 72 °C for 30 s. Following the first 15 cycles, 10 μM each of primers P1 and P6 were added to each reaction tube followed by 25 cycles of 95 °C for 30 s, 60 °C for 30 s, and 72 °C for 5 min, plus an increment of 30 s for each cycle. A final elongation step at 72 °C for 10 min was included. The PCR construct (expected size 1260 bp) was purified using QIAquick gel extraction kit (Qiagen^®^, Valencia, CA, USA).

### 4.3. Transformation

Competent cells of strain OKB105 were prepared according to the procedure of Cutting and Vander Horn [59]. To check for competence, OKB105 cells were transformed with the *rpoB* gene of the rifampicin-resistant *Bacillus mojavenesis* strain JF-2 [52,53]. DNA of *Bacillus mojavenesis* strain JF-2 *rif* ^r^ was extracted using QIAamp DNA Mini Kit (Qiagen^®^, Valencia, CA, USA), and the *rpoB* gene was amplified using primers rpoBF and rpoBR (Table 4). The reaction contained 2 μL of the extracted DNA, 1 × PCR buffer with magnesium sulfate (Roche^®^, Boulder, CO, USA), 0.2 mM dNTPs mixture, 0.5 U of the Expand Long Template PCR System Taq polymerase (Roche^®^, Boulder, CO, USA), and 10 μM each of the forward and the reverse primers. PCR protocol was an initial denaturation at 95 °C for 5 min followed by 35 cycles of denaturation at 95 °C for 1 min, annealing at 50 °C for 1 min, and elongation at 72 °C for 3 min. A final elongation step at 72 °C for 5 minutes was included. The PCR product (∼3.3 Kb) was purified using MinElute PCR purification kit (Qiagen^®^, Valencia, CA, USA) and used to transform OKB105 competent cells [59]. OKB105 competent cells were then transformed with the *ybdT*-*cat* PCR construct.

To induce the chloramphenicol-resistance genes, the transformed cells were added to antibiotic-free LB plates in an overlay of LB soft agar with 0.1 mL of 50 μg/mL chloramphenicol followed by a 2 h incubation at 37 °C. A second overlay of LB soft agar with 0.1 mL of 2 mg/mL chloramphenicol was then added and the plates were incubated at 37 °C for 1–2 days until colonies were visible [59]. Colonies were streaked onto blood agar plates to identify transformants that were non-hemolytic, indicating that they produced little or no biosurfactant. The *ybdT* gene was amplified from chloramphenicol-resistant, non-hemolytic mutants using primers P1 and P6 (Table 4). The PCR product was purified and sent for sequencing at the Oklahoma Medical Research Foundation. One chloramphenicol-resistant, non-hemolytic mutant (NHY1) with the chloramphenicol cassette inserted into the *ybdT* gene was characterized further.

### 4.4. Expression of srf (Surfactin Synthetase) Gene

Cell pellets of OKB105 (wild type) and NHY1 were first stabilized with RNAprotect Bacteria reagent (Qiagen^®^, Valencia, CA, USA) before RNA purification with RNeasy Protect Bacteria Mini kit (Qiagen^®^, Valencia, CA, USA). DNA was digested during RNA extraction with RNase-Free DNase set (Qiagen^®^, Valencia, CA, USA). RNA from both OKB105 and NHY1 were reverse-transcribed to cDNA using gene-specific primers P6 for *ybdT* and SrfR for *srf*1 using SuperScript II Reverse Transcriptase kit (Invitrogen^®^, Carlsbad, CA, USA) according to manufacturer’s instructions. The cDNA from OKB105 and NHY1 was then used in PCR reactions to amplify *ybdT* and *srf* genes. As a control, purified RNA was used as the template for the PCR reaction in the absence of reverse transcriptase to verify that the preparation did not contain DNA.

### 4.5. Assay of YbdT Activity

Cell-free extracts were prepared as described previously [54] with the exception that the ammonium sulfate fractionation step was omitted. The peroxygenase activity of YbdT was measured in cell-free extracts of OKB105 (wild type strain) as described before [39]. The reaction mixture (0.2 mL) contained 0.1 M potassium phosphate buffer (pH 5.9), 1.32 mM H_2_O_2_, and 60 μM myristic acid. The reaction was started by the addition of the cell free extract followed by incubation at 37 °C for 10 min. The reaction was stopped by the addition of 50 μL of 12 N HCl. The reaction mixture was extracted with 250 μL of ethyl acetate: hexane (1:1 v/v). The organic layer was evaporated and the residue was derivatized with N,O-bis(trimethylsilyl)trifluoroacetamide (BSTFA) (Pierce, Rockford, IL, USA) and analyzed by GC/MS (Agilent Technologies 6890N Network GC systems/5973 Network Mass Selective Detector, Willmington, DE, USA) [29]. One microliter was used for injection. The oven temperature was set at 60 °C for 5 min and then increased to 250 °C over a 15 min interval. The column was a capillary column 0.25 mm × 30 m × 0.25 μm. The carrier gas was helium and the flow rate was 1.2 mL/min. The mass spectrometer was operated at 400 Hz. Peak areas obtained on the GC chromatogram were used to calculate the percentage of the 3-hydroxy fatty acids compared to the area of all detected fatty acids. The mass spectrum at *m/z* ratio of 233, which is characteristic of bis-trimethylsilyl (TMS)-derivatized, 3-hydroxy fatty acids (Figure 2), was extracted. Bis-TMS derivatized 3-hydroxymyristic acid (Larodan Fine Chemicals^®^, Malamö, Sweden) was used as the standard. Protein concentrations were determined by the method of Bradford [60] using bovine serum albumin as a standard.

### 4.6. Biosurfactant Synthesis in Cell-Free Extracts from l-Amino Acids and 3-Hydroxymyristoyl-CoA

Cell-free extracts of NHY1 and OKB105 were prepared and subjected to ammonium sulfate fractionation as described previously [54]. Protein that precipitated between 30–70% saturation with ammonium sulfate was desalted and eluted in 3.5 mL volume using Amersham^®^ (GE Healthcare, Piscataway, NJ, USA) desalting columns PD10 according to the manufacturer’s instructions. Cell-free extracts were tested for the ability to synthesize surfactin [54].

The synthesis of surfactin from the l-amino acids and 3-hydroxymyristoyl-CoA in presence of Mg-ATP was performed in a 0.5 mL reaction mixture that contained: 10 mM ATP, 10 mM MgCl_2_, 13 mM dithioeryhthritol (DTE), 5 mM each of l-aspartate, l-glutamate, and l-valine, 0.5 mM l-leucine, 80 mM TrisHCl (pH 7.8), 0.1 mM of 3-hydroxymyristoyl-CoA, 1.3 mM potassium phosphate, and 1 mM ethylenediamine tetra-acetic acid (EDTA). The reaction was started by the addition of cell-free extract to give 20, 50, 100, 200, and 200 μg/mL, incubated at 37 °C for 60 min, and stopped by placing the mixture on ice [54]. The reaction was monitored by following the disappearance of the 3-hydroxymyristoyl-CoA, the release of CoA, and the production of the biosurfactant by high-pressure liquid chromatography (HPLC).

The concentration of 3-hydroxymyristoyl CoA was determined by using a HPLC equipped with an Alltech Prevail™ C18, 5 μm, 4.6 × 150 mm column (Grace Discovery Sciences, Deerfield, IL, USA), a mobile phase of 75% methanol and 25% 25 mM KH_2_PO_4_ (pH 5.3) and UV detection at 259 nm. The amount of 3-hydroxymyristoyl-CoA in the reaction mixture over time was determined by comparison of peak area to a standard curve prepared with known concentrations of 3-hydroxymyristoyl-CoA. Results were corrected for abiotic loss of 3-hydroxymyristoyl-CoA determined from controls that lacked cell-free extract.

The concentration of CoA in the reaction mixture over time was determined by HPLC with an Alltech Prevail™ C18, 5 μm, 4.6 × 150 mm column (Grace Discovery Sciences, Deerfield, IL, USA), a mobile phase of 5% acetonitrile in 25 mM KH_2_PO_4_ (pH 5.3), and UV detection at 259 nm. Peak areas were compared to a standard curve prepared with known amounts of CoA and corrected for CoA production in controls that lacked cell-free extract.

Biosurfactants in the reaction mixture were concentrated by precipitation with 40% ammonium sulfate overnight at room temperature followed by centrifugation and re-dissolving the pellet in a minimal volume of water. The purified biosurfactants were quantified by using HPLC with an Alltech Prevail™ C18, 5 μm, 4.6 × 150 mm column (Grace Discovery Sciences, Deerfield, IL, USA), a mobile phase of 70% acetonitrile, and 30% 25 mM KH_2_PO_4_ (pH 5.3), and UV detection at 210 nm. A standard curve of the commercially available surfactin (Sigma) was used to calculate the concentration of the biosurfactant in the reaction mixture compared to controls that lacked cell-free extracts.

### 4.7. Enzymatic Preparation of 3-Hydroxymyristoyl-CoA

Instead of the chemical synthesis used by Ullrich *et al.* [54] to synthesize 3-hydroxymyristoyl-CoA, we synthesized 3-hydroxymyristoyl-CoA enzymatically with an acyl-CoA synthase enzyme from *Pseudomonas* sp. available from Sigma^®^. This enzyme has been used before to synthesize CoA derivatives of dicarboxylic acids [61]. First, we determined that 3-hydroxymyristic acid (Larodan Fine Chemicals^®^, Malamö, Sweden) could be used as a substrate by the acyl CoA synthase. The reaction mixture contained: 50 mM TrisHCl (pH 8), 5 mM MgCl_2_, 390 μM NADH, 5 mM ATP, 0.38 mM CoA, 8 U of myokinase, 7.4 U of pyruvatekinase, 9.3 U of lactate dehydrogenase, 1.06 mM phosphoenolpyruvate, and 0.025 mg of 3-hydroxymyristic acid in a final volume of 0.6 mL. The reaction was started by the addition of 0.04 U of acyl-CoA synthase and incubated at room temperature for 5 min. The reaction was followed by measuring the loss of absorbance at 340 nm due to the oxidation of NADH coupled the production of AMP [62].

To synthesize 3-hydroxymyristoyl-CoA, 15 mg (60 μmol) of the free acid, 0.12 mmol of CoA, 0.54 mmol of ATP, and 1.92 U of acyl-CoA synthase were combined in a final volume of 50 mL of 50 mM Tris HCl and 5 mM MgCl_2_. The reaction was incubated at room temperature for 5 hours with low-speed stirring, after which an additional 0.12 mmol of CoA and 0.54 mmol of ATP were added and the reaction incubated at room temperature overnight [63].

### 4.8. Purification and Quantification of the 3-Hydroxymyristoyl CoA

Prevail™ 10 g-Solid-Phase-Extraction C18 columns (Grace Discovery Sciences, Deerfield, IL, USA) were used for the 3-hydroxymyristoyl-CoA purification. Columns were activated with methanol, followed by deionized water, and then 100 mM MOPS-NaOH buffer. The 50 mL reaction was loaded on the activated columns and the unreacted CoA was eluted using 50 mL of MOPS-NaOH, followed by 50 mL of 50% methanol until the absorbance at 260 nm of the eluant approached zero. The 3-hydroxymyristoyl-CoA was then eluted from the column using 100% methanol until the absorbance at 260 nm of the eluant approached zero. The 100% methanol fractions were combined and evaporated under N_2_ overnight. The residue was dissolved in 0.1 mM dithiothreitol (DTT) and stored at −70 °C until used [63]. The purity of the product was checked using HPLC with a Discovery RP-Amide C16 column (Supelco) (25 cm × 4.6 mm, 5 μm). The flow rate was 1 mL min^−1^ using 20 mM ammonium formate pH 5 with an initial concentration of 5% acetonitrile. After 5 min, the acetonitrile concentration was increased over a 10 min period to 15%. Peaks were detected at 254 nm and further monitored using a diode array detector to scan from 210 to 400 nm. Injection volume was 20 μL. To quantify the amount of 3-hydroxymyristoyl-CoA present, an aliquot was hydrolyzed in base by adjusting the pH to 10–11 with NaOH followed by incubation at room temperature for 30 min to release CoA, which was measured as described above.

### 4.9. Biosurfactant Synthesis by Cell-Free Extracts from l-amino Acids, Myristic Acid and CoA

Cell-free extracts of NHY1 and OKB105 were tested for the ability to synthesize surfactin from l-amino acids, myristic acid, and CoA in the presence of H_2_O_2_ (substrate for YbdT) and ATP/Mg. Cell-free extracts were prepared as described in Ullrich *et al.* [54] without the ammonium sulfate fractionation step. The reaction mixture (0.5 mL) contained 5 mM each of l-aspartate, l-glutamate, and l-valine, 0.5 mM l-leucine, 10 mM ATP, 10 mM MgCl_2_, 13 mM DTE, 6 mM myristic acid, 6 mM CoA, 1.32 mm H_2_O_2_, 1.3 mM potassium phosphate, 1 mM EDTA, and 80 mM Tris HCl (pH 7.8). The reaction was started by the addition of 100 μg of the cell-free extract. The mixture was incubated at 37 °C for 90 min and then stopped by placing the mixture on ice. The biosurfactant was precipitated overnight at room temperature with 40% ammonium sulfate [29]. The precipitate was dissolved in 100 μL water and quantified by HPLC as described above.

### 4.10. Determination of the Biosurfactant Structure

The amino acid and fatty acid compositions, as well as the molecular weight were used to compare the structures of the biosurfactants produced by NHY1 and OKB105. Cells were grown in 500-mL medium E and biosurfactants were purified as described before [29]. The amino acid composition was determined by using cation exchange chromatography at the molecular biology research facility of the William K. Warren Research Institute (Oklahoma City, OK, USA) as described before [29]. For fatty acid analysis, 200 μg of the purified biosurfactant was hydrolyzed under vacuum for 16 hours at 90 °C with minimal volume of 6 N HCl in sealed tubes. The hydrolyzed fatty acids were then extracted with 7 mL of 1:1 v/v ethyl acetate: hexane. The organic phase was concentrated under a stream of N_2_. The concentrated fractions were neutralized with 0.5 mL of 0.4 M phosphate buffer (pH 12) and incubated at room temperature for 10 min. The fatty acids in the organic layer were derivatized with BSTFA and analyzed by GC/MS. Methanolysis was conducted as described before [29]. To analyze the fatty acids, the mass spectrometer chromatogram was extracted at *m/z* ratio of 233 characteristic of bis-TMS derivatized, 3-hydroxy fatty acids, and at *m/z* ratio of 117 characteristic of TMS-derivatized, non-hydroxylated fatty acids (Figure 2). To analyze the fatty acid methyl esters, the mass spectrometer chromatogram was extracted at *m/z* ratio of 175 characteristic of TMS-derivatized 3-hydroxy fatty acid methyl ester, and at *m/z* ratio of 74characteristic of non-hydroxylated fatty acid methyl esters (Figure 2). Retention times and mass spectra were compared to authentic standards of methyl-3-hydroxytetradecanoic acid, 3-hydroxytetradecanoic acid, methyltetradecanoic acid, and tetradecanoic acid (Larodan Fine Chemicals^®^, Malamö, Sweden) derivatized as described above.

The molecular weights of biosurfactants purified from NHY1 and OKB105were determined with electrospray ionization mass spectrometry [7,47–49]. The biosurfactants were dissolved in 10 mM ammonium hydroxide to a final concentration of 0.05 μg/μL. The solvent system used was 75% methanol: 25% water. Samples were run in the negative mode.

## 5. Conclusions

An in-frame mutation of *ybdT* was obtained and phenotypic characterization of the mutant strain (NHY1) showed that YbdT catalyzes the formation of 3-hydroxy LCFA needed for lipopeptide biosurfactant synthesis in *B. subtilis*. NHY1 produced predominantly non-hydroxylated lipopeptide biosurfactants with diminished activity. Site-directed mutagenesis of the enzyme responsible for 3-hydroxylation of LCFA could provide a new avenue to design biosurfactants tailored for specific functions by altering the 3-hydroxy fatty acid composition of the biosurfactant.

## Figures and Tables

**Table 1.** Surfactin synthase activity in OKB105 and NHY1 cell-free extracts in the presence of 3-hydroxymyristoyl-CoA^a^.

**Cell-Free Extract**	**Coenzyme A Release (nmol·min^−1^·ng·protein^−1^)**	**3-Hydroxymyristoyl-CoA Consumption (nmol·min^−1^·ng·protein^−1^)**	**Biosurfactant Production (ng·min^−1^·ng·protein^−1^)**
OKB105	0.018 ± 0.015 ^b^	0.002 ± 0.002 ^b^	17.4 ± 5.5 ^b^
NHY1	0.018 ± 0.017	0.001 ± 0.001	17.0 ± 1.9

Student’s *t*-test *p*-value	0.98 ^c^	0.23 ^c^	0.51 ^c^

a Each product was detected and quantified by high-pressure liquid chromatography. Standard curves were constructed using coenzyme A, enzymatically synthesized 3-hydroxymyristoyl-CoA, and commercially available surfactin (Sigma^®^);

b Values are means ± standard deviations of 9 measurements from 2 separate experiments for OKB105 extracts and 10 measurements from 2 separate experiments for NHY1 extracts for coenzyme A release and 3-hydroxymyristoyl CoA consumption assays. For biosurfactant production, the values are means ± standard deviations of 3 measurements for each extract;

c The *p*-value associated with Student’s *t*-test [42] was used to test the significant difference between enzyme activities of OKB105 and NHY1 extracts.

**Table 2.** Fatty acid composition of OKB105 and NHY1 biosurfactants.

**Fatty Acid (FA) Chain Length**	**OKB105 Biosurfactant Fatty Acid Composition**				**NHY1 Biosurfactant Fatty Acid Composition**			

	**Non-Hydroxylated FA**		**3-Hydroxylated FA**		**Non-Hydroxylated FA**		**3-Hydroxylated FA**	

	**Methanolysis ^a^**	**Acid Hydrolysis ^a^**	**Methanolysis**	**Acid Hydrolysis**	**Methanolysis**	**Acid Hydrolysis**	**Methanolysis**	**Acid Hydrolysis**
12	ND ^c^	ND	ND	ND	8.61 (1) ^b^	21.2 (1)	ND	ND
13	11.6 (1)	ND	10.26 (3)	1.87 (2)	13.06 (1)	ND	1.99 (1)	ND
14	18.23 (2)	6.18 (2)	5.15 (2)	0.63 (1)	1.92 (1)	12.6 (2)	3.57 (1)	2.18 (2)
15	61 (1)	19.9 (2)	31.3 (3)	8.9 (2)	ND	5.46 (2)	7.28 (2)	ND
16	3.42 (1)	32.6 (2)	ND	ND	25.3 (3)	36.2 (2)	ND	ND
17	ND	10.1 (1)	ND	ND	ND	4.19 (1)	ND	ND
18	26.2 (2)	25.3 (2)	ND	ND	60.1 (3)	31 (2)	ND	ND
Total	66.6 (3)	55.4 (2)	33.4 (3)	44.6 (2)	93.3 (3)	97.8 (2)	6.72 (3)	2.18 (2)
Avg.	60.97		39		95.6		4.4	

a Methanolysis and acid hydrolysis refer to the method used for hydrolysis of the biosurfactant fatty acid;

b Numbers are the percentage of the fatty acid calculated from the peak areas of gas chromatogram. Numbers in parenthesis are the number of times the isomer was detected. Three methanolysis and two acid hydrolysis preparations were analyzed;

c ND: not detected.

**Table 3.** Molecular weights of OKB105 and NHY1 biosurfactants determined by electrospray ionization mass spectrometry.

**Deduced FA Chain Length**	**OKB105 Biosurfactant ^a^**			**NHY1 Biosurfactant ^a^**		

	**M + H^+ b^**	**M + Na^+ b^**	**M + 2Na^+^ − H^+ b^**	**M + H^+ b^**	**M + Na^+ b^**	**M + 2Na^+^ − H^+ b^**
11			1024		**1004**	
12		**1018**	**1040**		1016, **1018**	
13	1008	**1032**			1030, **1032**	
14	1022	1044			1044, **1046**	
15	1036	1058				
16	1050	1072		**1052**		

a Biosurfactants from the OKB105 and NHY1 cultures were purified and subjected to electrospray ionization mass spectrometry in the negative mode;

b Numbers are the molecular mass of the detected ionized species as indicated in the column header. Numbers in bold refer to molecular weights that are +2 mass units greater than the corresponding 3-hydroxy fatty acid and indicate a non-hydroxylated acyclic structure. Peaks with mass units 1018, 1040, and 1032 in the OKB105 biosurfactant corresponding to the non-hydroxylated acyclic isomers had intensities ranging from 800 to 1150 counts compared to intensities ranging from 850 to 2400 counts for the other peaks. Also, peaks with mass units 1016, 1030, and 1044 in the NHY1 biosurfactant corresponding to the hydroxylated cyclic isomers had intensities ranging from 850 to 1200 counts compared to intensities ranging from 1000 to 5900 counts for the other peaks.

**Table 4.** Primers used in this study.

**Primer**	**Sequence (5′-3′)**
P1F ^a^	ATGAATGAGCAGATTCCACATGACAAAAGT
P2R ^a^	TGCCTCCTA*ACCTGCCCA*ATAGCACGCTACCCG
P3F ^a^	*TGGGCAGGT*TAGGAGGCAATGAACTTTAAT
P4R ^a^	AATGCCTTC*TAAAAGCCA*GTCATTAGG
P5F ^a^	*TGGCTTTTA*GAAGGCATTACAATTGAAGTCAT
P6R ^a^	ACTTTTTCGTCTGATTCCGCTCATTACGAA
Srf1F ^b^	GCGGTAGAAAAACTGCTTGC
Srf1R ^b^	ACAGGTTCGTCTGCTTTGCT
rpoBF ^c^	TCAACTAGTTCAGTATGGACG
rpoBR ^c^	ACCTGGTTCAGGAACATTGTC

a Primers P1–P6 were used for the PCR construct. P1 and P2 amplify the 5′ region of *ybdT* gene, P5 and P6 amplify the 3′ region of the *ybdT* gene, and P3 and P4 amplify the chloramphenicol resistance cassette. Underlined regions and italicized regions in P2 and P3 and in P4 and P5 are complementary and are used to create the 18 bp overlap in each case;

b Primers Srf1F and Srf1R amplify a 250 bp region of surfactin synthesis (*srf*) operon;

c Primers rpoBF and rpoBR amplify a 3.3 Kb region of *rpoB* gene.
